# Chiropractic, one big unhappy family: better together or apart?

**DOI:** 10.1186/s12998-018-0221-z

**Published:** 2019-02-21

**Authors:** Charlotte Leboeuf-Yde, Stanley I. Innes, Kenneth J. Young, Gregory Neil Kawchuk, Jan Hartvigsen

**Affiliations:** 1 Institute for Regional Health Research, University of Southern Denmark, DK-5000 Odense C, Denmark; 2 School of Health Professions, Murdoch University, Murdoch, Australia; 3 Department of Physical Therapy, Faculty of Rehabilitation Medicine, University of Alberta, Corbett Hall, 8205 114 St NW, Edmonton, AB T6G 2G4 Canada; 4 Department of Sports Science and Clinical Biomechanics, University of Southern Denmark, Campusvej 55, 5230 Odense M, Denmark; 5 Nordic Institute of Chiropractic and Clinical Biomechanics, Campusvej 55, 5230 Odense M, Denmark

**Keywords:** Allied health, Attitude of health Personnel, Chiropractic, Professionalisation, Social perception, Trends

## Abstract

**Background:**

The chiropractic profession has a long history of internal conflict. Today, the division is between the ‘evidence-friendly’ faction that focuses on musculoskeletal problems based on a contemporary and evidence-based paradigm, and the ‘traditional’ group that subscribes to concepts such as ‘subluxation’ and the spine as the centre of good health. This difference is becoming increasingly obvious and problematic from both within and outside of the profession in light of the general acceptance of evidence-based practice as the basis for health care.

Because this is an issue with many factors to consider, we decided to illustrate it with an analogy. We aimed to examine the chiropractic profession from the perspective of an unhappy marriage by defining key elements in happy and unhappy marriages and by identifying factors that may determine why couples stay together or spilt up.

**Main body:**

We argue here that the situation within the chiropractic profession corresponds very much to that of an unhappy couple that stays together for reasons that are unconnected with love or even mutual respect. We also contend that the profession could be conceptualised as existing on a spectrum with the ‘evidence-friendly’ and the ‘traditional’ groups inhabiting the end points, with the majority of chiropractors in the middle. This middle group does not appear to be greatly concerned with either faction and seems comfortable taking an approach of ‘you never know who and what will respond to spinal manipulation’. We believe that this ‘silent majority’ makes it possible for groups of chiropractors to practice outside the logical framework of today’s scientific concepts.

**Conclusion:**

There is a need to pause and consider if the many reasons for disharmony within the chiropractic profession are, in fact, irreconcilable. It is time to openly debate the issue of a professional split by engaging in formal and courageous discussions. This item should be prioritised on the agendas of national associations, conferences, teaching institutions, and licensing/registration as well as accreditation bodies. However, for this to happen, the middle group of chiropractors will have to become engaged and consider the benefits and risks of respectively staying together or breaking up.

## Background

### Health care is becoming increasingly evidence-based

Over the past decades, governments, society and patients have an increasing expectation of an evidence-based approach to health care and as the knowlege base has become larger and more widely accepted, the space available for alternative modalities has become smaller [1–4]. This has resulted in a greater contrast between mainstream and fringe medicine. Also, in the musculoskeletal area there are now different demands on indications for treatment and positive outcomes than what was seen only a few decades ago [5]. Increasingly legislation is being bought to bear to enforce such approaches. Chiropractors have for many years balanced at the crossroads between mainstream and alternative medicine, so this development poses particular challenges for chiropractic organisations, who have tried to cater for both [6–8]. Although chiropractors, officially, are part of the evidence-based movement in relation to musculoskeletal problems, we were late adopters, and some are not prepared to adopt this approach at all.

### The consequences for chiropractors

To the public, chiropractors are known to be ‘back pain clinicians’ [9–12]. This is potentially a good niche, because back pain is one of the lagest public health problems, negatively affecting hundreds of millions of people. The number of years people live with disability has increased globally by 52% since 1990 [13, 14].The recent Lancet Low Back Pain Series pointed to the gap between what is known and what is being practiced, and called for a de-medicalisation of back pain and promotion of public health approaches in order to reverse this trajectory [15, 16]. Chiropractors appear to be well placed to respond to this call and become relevant in mainstream healthcare globally.

### Divisions within the chiropractic profession

As is the case with professions generally, chiropractic has always had subgroups, some further toward the fringe and others closer to mainstream healthcare. Chiropractic arose out of a vitalistic tradition. D.D. Palmer, the founder of chiropractic, declared that 95% of all disease was caused by subluxated vertebrae and that the remaining 5% was caused by slight displacements of bones other than those in the spine [17]. An early split developed between the vitalists and those who developed towards a more scientific approach, and divisions remain. It is estimated that approximately 20% of the profession in Canada still adheres to a vitalistic explanation for how they practice [18, 19]. Despite the presence of these factions, chiropractic has gradually become a global healthcare profession [20], and in some jurisdictions chiropractors are regarded as mainstream healthcare providers, as part of national health systems or reimbursed with public or private insurance funds. In places where chiropractors have adopted modern evidence-based principles, external stakeholders have determined that chiropractic practice accords with modern healthcare principles and should be included among legitimate healthcare practitioners [21, 22]. Nevertheless, there is a continuing divide between ‘evidence-friendly’ and ‘traditional’ chiropractors, which has become more visible in recent years, as the focus on back pain and musculoskeletal health has increased and a wealth of new evidence in the area has emerged. Unfortunately, these disparate voices reflecting different approaches confuse external stakeholders and threaten the credibility of the chiropractic profession.

Describing this division is not simply academic; those aligned with evidence-based ideals have the greatest probability of being further integrated into healthcare systems in the years to come. In contrast, chiropractors, who have traditional ideas of the spine being a source of all or most diseases, are unlikely to make this journey, in the absence of evidence to substantiate their claims. The result is a profession torn between those looking to the future and those wedded to the founding claims of the past. The evidence-friendly chiropractors feel that the claims and activities by the ‘traditionalists’ slows or hinders the development of the profession and there is evidence to substantiate this.

Here are some examples of this problem, as seen from the perspective of the evidence-friendly group:

- In Canada, vitalist practitioners have been shown to be more likely to have anti-vaccination beliefs, and their attitudes about radiographic imaging are inconsistent with current evidence/ guideline-based care [19]. As such, vitalistic providers were less likely to receive referrals from other health providers [23].
- In Florida, U.S.A., attempts to establish a university-based education in chiropractic were stopped in 2005 because of opposition and lobbying from the traditional group [24].
- In 2009 in the UK, a systematic survey of chiropractic websites was done by a group motivated by displeasure at unsupported claims of chiropractors, and formal complaints were lodged with the General Chiropractic Council. Although most chiropractors were found not guilty, thousands of work-hours and much stress was caused [25, 26]. The content of these web sites was subsequently changed.
- In 2012, the treatment of children based on traditional chiropractic ‘diagnoses’ at the student chiropractic clinics at the Royal Melbourne Institute of Technology in Australia, a university-based chiropractic course, at the time lead by a well-known traditionally-oriented chiropractor, brought down both fury and ridicule on chiropractic. It also resulted in a new movement called ‘Friends of Science’, who wage war on university education involving non-evidence-based alternative medicine, notably chiropractic. This severely threatened at least two chiropractic undergraduate courses [27, 28].
- In 2013, attempts to establish a university-based education in chiropractic in Sweden were stopped following a debate that exposed unsupported claims on the websites of some chiropractors [29].

There is also evidence that traditional chiropractors feel aggrieved by the evidence-friendly group, because they think that ‘real’ chiropractic is being denigrated or squandered.

And here are some examples of the problem as seen from the perspective of the traditional group:

- Evidence-friendly chiropractors are seen as unfaithful to the traditional tenets of chiropractic (i.e. subluxation as a basis for changes in health).
- Evidence-friendly chiropractors side with the ‘enemy’, i.e. medical doctors, research scientists, sceptics, etc.

Evidence for this is extensive, but a few examples from current chiropractic websites include the following:

- ‘Straight [traditional] chiropractors consider the medical diagnosis of disease to be unnecessary because they view these conditions as secondary effects of vertebral subluxations. In essence, they believe that disease symptoms will disappear once the underlying subluxations have been corrected properly. Most straight chiropractors do not wish to have any association with mainstream health care’ [30].
- ‘Chiropractic’s new normal should look a lot like its old normal. You know, the one centered on our only unique service to mankind: the detection, correction and management of vertebral subluxation’ [31].
- ‘If you choose the more allopathic path of chiropractic medicine, your practice style will be different. You’ll befriend orthopedists and other medical practitioners, supplying a form of physical medicine. You’ll add various therapies, decompression, orthotics, stretches, exercises, rehab and other adjunctive services to support your spinal manipulations. ……you will be reduced to proper lifting, pillow recommendations and maybe even weight loss’ [32].

Given the slates of problems observed by these two groups, the question for the profession to face is, what can be done about it?

### One solution: Trying to unite a divided profession

In response to this division, various chiropractic organisations have since long made great efforts to try to bring the various factions closer together by appreciating and respecting each other’s differences. One such unifying attempt from the European Chipractors’ Union (ECU) was the slogan ‘celebrating diversity’ used in connection for the 2016 ECU conference [33]. However, it is not evident that this diversity should be celebrated. We do not see ‘diversity’ as a strength for either of the two main factions of chiropractors, or more importantly for patients, who must employ a caveat emptor approach to finding a chiropractor.

A more recent approach that we are seeing now is that various organisations, who for decades have supported unity between factions at all costs, are now focusing strongly on evidence as the basis for chiropractic practice rather than on unity. One example is the American Chiropractic Association (ACA) that recently adopted the mainstream, broad-consensus ‘Choosing Wisely’ campaign, and the World Federation of Chiropractic (WFC) that recently signaled a policy shift by abandoning attempts at ‘unity’ [7] and focusing on ‘on the creation of trust, legitimacy and promoting the best available care’ [34, 35]. This tactic changes the focus from the chiropractor, to where the focus of any legitimate healthcare profession should be, on the patient. The end results should, hopefully, be a general shift towards a more modern approach and away from old traditions.

### Another solution: Creating a permanent division through “professional divorce”

In this commentary, we explore another solution to this historic division. One that does not involve uniting both sides, but one that acknowledges that when a division is too great to reconcile, decoupling may be the best way forward for all involved. In this paper, we explore the similarities between couples and professions, examine what happens when they become estranged, and consider how the solutions established by our society for marital disagreement may also be the key to doing what is best for both groups. Uppermost in our minds in this process is what is best for the ‘children’, i.e. patients.

### What makes spouses happy, or at least content, in their marriages?

Obviously, there are many and varied reasons for making married couples happy and functional. Love, affection, and sex [36] are important initiators for many marriages and helpful to maintain the relationship also in the long run. To produce, provide for, and ensure the survival of offspring is another uniting aspect [37]. A feeling of togetherness, defined as easy communication, similar habits, hobbies, activities, and the ability for constructive problem-solving makes living together pleasant, as does a common history, and a similar background [36, 38]. Solidarity provides a strong cornerstone in successful marriages [39], as does the concept of being a happy family and the status associated with this in society [40]. Clearly, being in a stable financial situation would help make a couple more trouble-free and hence invite fewer problems and result in less risk of disenchantment and disagreements that potentially can lead to a split [41, 42].

### Why may couples stay together when things go bad?

These reasons for content marriages are hardly surprising. However, why some couples choose to stay together, when the relationship has gone sour and there is no love left between the parties, is relevant to this discussion.

Respect for each other, morality, religion, politics, and business interests may be some reasons for remaining, as well as the wish not to hurt the other partner [37]. Some prefer to wait ‘till the children have grown up’ [39] and in others it is the fear of the unknown including concerns about potential economic hardship [42]. It is also well known that feelings and relationships can have their ups and downs, so some may simply ‘hang around’ hoping for better times [41].

Additionally, when marriages are clearly dysfunctional and the two partners would be better off on their own, it is not uncommon for one of the two to make promises to change (e.g. no more extra-marital sex, more time spent with the family, no more fighting, no more violence) [43, 44]. Kindness, wishful thinking, memories of happy early days, and financial weakness could also be reasons why the aggrieved partner may give the marriage another chance, and perhaps another and another.

Finally, when things go wrong in a marriage, another strategy is that one partner has too much to lose so they simply tolerate the problem – perhaps this is the closest analogy of where the chiropractic profession has been in the past 25 years. Doubtlessly though, both parts are unhappy with the present arrangement.

### Why may couples choose to divorce?

Instead of choosing to remain in a relationship for any number of reasons, there are obviously many couples who decide to separate [45, 46]. The reasons for divorce have been a major topic of research and have been comprehensively reviewed elsewhere [46]. This review summarises longitudinal studies that identify the predictors of marital disruption as being domestic violence, frequent conflict, and infidelity, the number of perceived relationship problems, and low levels of love, trust and commitment between spouses. Minimising the difficulties, confronting relationships by using benevolent cognitions such as ‘better the devil you know’ only allows relationships to worsen over time [47]. Although divorces are always difficult, the outcome is often better for both partners in the long run, offering possibilies for a more self-actualised existence [48–50].

### Signs that the chiropractic profession is an unhappy marriage

The two main factions in the chiropractic profession still ‘live at the same address’. By this we mean that they present themselves to others under the same family name, have institutions that try to enforce the same international and national Standards of Accreditation of chiropractic programmes for both so that patients are dealt with in fairly standardised ways, and there are regulations for chiropractic educations to ensure a reasonable common level of graduates.

Nevertheless, there are definite signs that the situation may be intolerable for many chiropractors on both sides. We have identified reasons for unhappiness, and listed those in Table 1. These reasons are based on our observations of what happens in the field and are thus personal opinions, not easily documented from scientific evidence. Therefore, the items listed have not been referenced. Nevertheless we believe that most of our colleagues will acknowledge these issues and that they resemble very much those described above in reasons for divorce among couples.

**Table 1:** A list of signs of incompatibility between the evidence-friendly and traditional factions in chiropractic, described as it would be in an unhappy marriage, as seen from the evidence-friendly view point

Important ingredients in a marriage	Signs of unhappiness in the chiropractic ‘marriage’ between evidence-friendly and traditional chiropractors
Love	• It is evident that there is no love between two groups. Neither wishes to spend time or more intimate moments with the other.
Respect	• There is little tolerance between the two factions.
Agreement on common basic concepts	• The evidence-friendly group adopt a natural sciences critical thinking approach and more easily accepts good quality scientific studies, regardless the results, while the others disregard evidence, if it does not confirm their prior beliefs. Traditional chiropractors are also prepared to accept substandard research such as case-reports as evidence.
Easy communication; togetherness; similar interests	• The two factions find it difficult to communicate because the evidence-friendly groups seek to use contemporary mainstream language, whilst the others stick to traditional language, e.g. ‘subluxation’, ‘innate intelligence’, ‘adjustment’, ‘the power that made the body heals the body’, and ‘treat the cause not the symptoms’. • There is no problem-solving mechanism. Therefore, central collaborative problems will rarely be discussed in order not to rock the boat too much. • Explanations to the patients about illness and health are different in the two camps; therefore it is difficult to exchange patients.
Extra-marital sex / infidelity	• The evidence-friendly chiropractors are seen as unfaithful by the traditionalists, as they ‘sleep’ with or have been seduced by members of external conventional health professions such as medicine and physiotherapy.
Intellectual differences	• The two groups do not attend the same type of seminars or conferences nor sit at the same table, when they are in the same room. • The improved status of the chiropractic profession depends largely on their participation in producing new evidence and this is done by the evidence-friendly group. • Very little of the research produced so far has succeeded in showing that treatment by chiropractors is superior to that delivered by other healthcare professions. Although the evidence-friendly group finds this disappointing, they maintain a patient-centered focus, confident that acceptance of truth is a necessary path to the best treatment options for patients as well as to mainstream acceptance of chiropractic methods. The traditional chiropractors seem unable to come to terms with this situation and build their professional activities and discourse on idealistic assumptions that either cannot be tested scientifically or are based on outdated health care models.
Disrespect, rudeness and nastiness	• Virulent attacks in social media are apparent when a scientific publication produced by evidence-friendly members fail to ‘prove’ what the traditionalists consider obvious, because they see proofs of this “every day in their clinics”.
Economic situation	• Evidence-friendly chiropractors are concerned about the traditional chiropractors’ exaggerated claims about cures, prevention and even longevity, which they consider deceptive. They think that biologically implausible claims and the resultant practice behaviours will have or have already affected the economic situation of the chiropractic family by creating distrust of the public and limiting the growth of the profession (i.e., the proportion of people seeking care).

### Why then are these two groups still joined together in their professional marriage?

Although there are many indications of unhappiness and also great attempts to improve the chiropractic marriage, no obvious signs of a *formal* splitting up are visible. Nevertheless, there are many reasons for some marriages to persist despite obvious difficulties and differences. The reasons for the chiropractic profession staying together may be analogous, and, as seen from our viewpoint, some of these are listed in Table 2 below:

**Table 2:** Possible reasons for the continued marriage between evidence-friendly and traditionalist chiropractors

Solidarity	• In front of authorities, the two factions often have to work together in order to appear to be a large and united profession, for which reason they attempt to reach a common ground, for example, when ‘officially’ defining chiropractic. Thus broad nebulous terms such as ‘prevention’, ‘spinal health’, ‘patient centered’ are used to prevent complete insight into what is really going on. • The past history (i.e. the concept that they have had a hard time, that they have fought together, and the feeling that they are ‘special’) seems to hold the two factions together against common ‘enemies’. • Personal friendships, often dating from formative years, also make it difficult to confront colleagues who we consider practising too much on the fringes of credibility.
Economy	• The evidence-friendly group has a financial interest in not being associated with the traditional chiropractors. However the latter group is riding on the credibility provided by the evidence-friendly group, which allows participation in legislation and reimbursement schemes and educational acceptance for their schools. • Being a large group rather than two smaller groups makes it easier to negotiate and deal with insurance agencies, government regulators, and health authorities.
Happy family	• Chiropractors have, traditionally, been socially isolated in the health care community but over the past decades the evidence-friendly chiropractors have been made welcome to join forces with traditional health care practitioners such as medicine and physiotherapy. However, when this occurs, the traditionalists are discretely kept in the background, not to scare policy makers and other stake holders off, which could stop this development.
Fear of unknown, weaker partner	• To separate the chiropractors into two professions would entail many changes, new political fights and an important task in relation to information and branding that doubtlessly requires careful consideration and a lot of work. • Clearly, the traditionalist group is most vulnerable because they would have to assume their true nature. Their approach is unlikely to appeal to authorities, third party payers and a large portion of the general public.
Hope of future improvements	• Many chiropractors do not see the problem as permanent, but view it as more of a short-lasting challenge, thinking that it is better to stay together, in order to be a large group and to have an influence on ‘the fringes’. • Many believe that extremists on both sides in the end will get to see the light and come and join ‘the middle ground’.
Family name	• Finally and very importantly, the family name (“chiropractor”) is central. To find another name would mean re-establishing connections with the public, insurance agencies, and government regulators and health authorities, which would not be easy. Therefore, both groups may well be hanging in there, mainly, in order to keep the name.

### The ‘middle group’

Chiropractors sit on a spectrum, and the majority fall in the middle and practice with a ‘you-never-know who and what will respond to spinal manipulation’ attitude, yet with only some attachments to chiropractic tradition. Chiropractors in this group probably just want to get on with their work, not paying too much attention to the bickering going on. They do not engage politically, they rarely appear at seminars or general assemblies, and they do not take sides. Thus this middle group accepts or at least tolerates much of the statements and activities by the traditionalist groups. Importantly, the middle group does not seem to consider illogical and unsubstantiated claims to be of real danger to the profession and if they are troubled by them, they do not voice this publically, maybe because they think that ‘the more the stronger’ or simply because of apathy.

In our opinion, this middle ground is becoming increasingly harder to reconcile and thus difficult to hold, as the marital difficulties inevitably play out more openly, because of the increasing general public interest in chiropractic [51–54]. Therefore, we contend that members of the middle group will eventually have to choose sides between adhering to a scope of evidence-friendly practice relating to musculoskeletal problems or to a traditional approach aiming at treating a multitude of conditions through spinal manipulation. Increasingly, this position reminds us of the saying ‘The standard you walk past is the standard you accept’, since acquiescence makes it possible for groups of chiropractors to practice outside the logical framework of today’s scientific concepts and sometimes even outside the law. Virtually every chiropractor knows of other chiropractors who x-ray every patient, or sign patients up to long contracts, or dubious ‘family plans’, or pre-payment plans, or use high-pressure sales tactics, or advertise unsubstantiated claims, yet very few report these breaches [55]. This ‘silent majority’ may in fact be responsible for the inertia and acceptance of traditionalist paradigms (seen from the evidence-friendly side) and the gradual destruction of traditional values (seen by the traditional chiropractors).

Chiropractors are not the only family in the neighbourhood. There are other families i.e., other manual therapeutic professions, who are engaged in ongoing positive positioning, who are willing, able and evidence centred to fill the societal need for conservative approaches to musculoskeletal care. It is likely that this time window of opportunity for chiropractors is limited and closing. Much like climate change perhaps the tipping point has already been breached. The time for action may never be more appropriate than now.

## Conclusions and perspectives

We acknowledge that vitalism and other idealistic concepts based on theories and beliefs rather than scientifically accepted logic and evidence have a role to play in the public domain. They are, however, not compatible with the ‘official’ evidence-friendly chiropractic profession, and to accept and protect such an approach, is a serious issue, potentially one of public safety.

We have argued that the situation within the chiropractic profession corresponds very much to that of an unhappy couple that stays together for reasons that are unconnected with love or even mutual respect. The current marital disharmony clearly goes beyond the scope of continuing to live unhappily with ‘another person’ of a differing world view. The alternative to this unhappy family structure would be an amicable divorce.

Although it might be painful, difficult and unsettling, in the long run it might make it possible for the two main groups to develop their full potentials, as they both deserve a happy professional life. The evidence-friendly group would be free to progress and collaborate in agreement with developing research findings and trends within public health, whereas the traditional groups can flourish in the wellness market, as there is a demand in the public also for more ‘mysterious’ and all-encompassing therapies and movements. For us it appears obvious that stakeholders, public, and the chiropractors would be better off, if the two factions and the middle group clearly stated where they belong. In addition, if patient interests are given true primacy, the arguments for unity, in our opinion, seem less significant than those for divorce.

Therefore, chiropractors and chiropractic leaders, regardless of values and persuasion, need to pause and consider, if they are able to live and develop as they would like to in this century-old unhappy marriage.

It is hoped that this paper opens a discussion among all parties that can eventually lead to an equitable arrangement for stakeholders and a sustainable future for chiropractic.

## Abbreviations

ACA: American Chiropractic Association
ECU: European chiropractic union
WFC: World Federation of Chiropractic

## References

[1] Murdoch B, Carr S, Caulfield T. Selling falsehoods? A cross-sectional study of Canadian naturopathy, homeopathy, chiropractic and acupuncture clinic website claims relating to allergy and asthma. BMJ Open. 2016;6(12). 10.1136/bmjopen-2016-014028 PMC5168671 27986744

[2] Johnson SM, Kurtz ME (2002). Perceptions of philosophic and practice differences between US osteopathic physicians and their allopathic counterparts. Soc Sci Med.

[3] Davidovitch N, Johnson RD (2004). Negotiating Dissent: Homeopathy and anti-vaccinationism at the turn of the Twentieth Century. The Politics of Healing: Histories of Alternative Medicine in Twentieth-Century North America.

[4] Kirschmann AT, Johnson RD (2004). Making friends for "pure" homeopathy: Hahnemannians and the Twentieth-Century preservation and transformation of homeopathy. The Politics of Healing: Histories of Alternative Medicine in Twentieth-Century North America.

[5] Brindle M, Goodrick E (2001). Revisiting maverick medical sects: the role of identity in comparing homeopaths and Chiropractics. J Soc Hist.

[6] Meeker WC, Haldeman S (2002). Chiropractic: a profession at the crossroads of mainstream and alternative medicine. Ann Intern Med.

[7] Identity Of The Profession [https://www.wfc.org/website/index.php?option=com_content&view=category&layout=blog&id=64&Itemid=93&lang=en].

[8] Bezold C, Thompson T, Arikan Y, Grandjean M (2013). Chiropractic 2025: divergent futures.

[9] Christensen MG, Hyland J, Goertz C, Kollash MG (2015). Practice analysis of chiropractic. National Board of chiropractic examiner.

[10] Wilson K, Swincer K, Vemulpad S (2007). Public perception of chiropractic: a survey. Chiropractic J Aust.

[11] Tsang VHM, Lo PHW, Lam FT, Chung LSW, Tang TY, Lui HM, Lau JTG, Yee HF, Lun YK, Chan HT *et al*. Perception and use of complementary and alternative medicine for low back pain. J Orthop Surg (Hong Kong). 2017;25(3):1–8. 10.1177/2309499017739480 29157106

[12] Brown BT, Bonello R, Fernandez-Caamano R, Eaton S, Graham PL, Green H (2014). Consumer characteristics and perceptions of chiropractic and chiropractic services in Australia: results from a cross-sectional survey. J Manip Physiol Ther.

[13] DALYs GBD, Collaborators H (2017). Global, regional, and national disability-adjusted life-years (DALYs) for 333 diseases and injuries and healthy life expectancy (HALE) for 195 countries and territories, 1990-2016: a systematic analysis for the global burden of disease study 2016. Lancet.

[14] Hartvigsen J, Hancock MJ, Kongsted A, Louw Q, Ferreira ML, Genevay S, Hoy D, Karppinen J, Pransky G, Sieper J (2018). What low back pain is and why we need to pay attention. Lancet.

[15] Buchbinder R, van Tulder M, Öberg B, Costa LM, Woolf A, Schoene M, Croft P, Hartvigsen J, Cherkin D, Foster NE. Low back pain: a call for action. Lancet. 2018;391(10137):2384–2388. 10.1016/S0140-6736(18)30488-4 29573871

[16] Foster NE, Anema JR, Cherkin D, Chou R, Cohen SP, Gross DP, Ferreira PH, Fritz JM, Koes BW, Peul W. Prevention and treatment of low back pain: evidence, challenges, and promising directions. Lancet. 2018;391(10137):9–15. 10.1016/S0140-6736(18)30489-6 29573872

[17] Palmer DD (1910). The Chiropractor's Adjuster: Textbook of the Science, Art and Philosophy of Chiropractic for Students and Practitioners.

[18] Puhl AA, Reinhart CJ, Doan JB, McGregor M, Injeyan HS (2014). Relationship between chiropractic teaching institutions and practice characteristics among Canadian doctors of chiropractic: a random sample survey. J Manip Physiol Ther.

[19] McGregor M, Puhl AA, Reinhart C, Injeyan HS, Soave D (2014). Differentiating intraprofessional attitudes toward paradigms in health care delivery among chiropractic factions: results from a randomly sampled survey. BMC Complement Altern Med.

[20] The Current Status of the Chiropractic Profession. Report to the World Health Organization from the WFC. [https://www.wfc.org/website/images/wfc/WHO_Submission-Final_Jan2013.pdf].

[21] Myburgh C, Hartvigsen J, Grunnet-Nilsson N (2008). Secondary legitimacy: a key mainstream health care inclusion strategy for the Danish chiropractic profession?. J Manip Physiol Ther.

[22] Schneider M, Murphy D, Hartvigsen J (2016). Spine care as a framework for the chiropractic identity. J Chiropr Humanit.

[23] Blanchette MA, Rivard M, Dionne CE, Cassidy JD. Chiropractors' characteristics associated with physician referrals: results from a survey of Canadian doctors of chiropractic. J Manip Physiol Ther. 2014;38(6):395–406. 10.1016/j.jmpt.2014.11.001 25939556

[24] Board of Governors kills FSU chiropractic school [https://www.heraldtribune.com/news/20050127/board-of-governors-kills-fsu-chiropractic-school].

[25] Brown R. Chiropractors: clarifying the issues. BMJ. 2009;339.

[26] Omnibus Complaint to the General Chiropractic Council [https://www.zenosblog.com/2009/06/omnibus-complaint-to-the-general-chiropractic-council/].

[27] Arndt B (2012). Universities are no place for quack medicine. The Australian.

[28] Recent Controversies in Chiropractic and RMIT Courses/Clinic [https://vicskeptics.wordpress.com/2011/09/29/recent-controversies-in-chiropractic-and-rmit-coursesclinic/].

[29] Brown R (2013). The status of chiropractic in Europe: a position paper. ECU.

[30] What’s the Difference Between a “Straight” Chiropractor and a “Mixer”? [http://www.gutierrezchiropractic.com/whats-the-difference-between-a-straight-chiropractor-and-a-mixer/].

[31] Chiropractic's identity problem. Our new normal. [https://chiropracticis.com/chiropractic-identity-problem/].

[32] Chiropractic vs. Chiropractic Medicine [https://www.patientmedia.com/chiropractic-vs-chiropractic-medicine].

[33] ECU Convention 2014 [http://www.chiropractic.ie/news-and-events/more/ECU-Convention-2014].

[34] Donohue A (2017). American Chiropractic Association releases Choosing Wisely list of tests, procedures to question.

[35] Tassel L. Trust and legitimacy are keys to advancement. In: World Federation of Chiropractic Quarterly Report. July 2018 edn. Toronto: World Federation of Chiropractic; 2018: 2–3.

[36] Johnson MD, Cohan CL, Davila J, Lawrence E, Rogge RD, Karney BR, Sullivan KT, Bradbury TN (2005). Problem-solving skills and affective expressions as predictors of change in marital satisfaction. J Consult Clin Psychol.

[37] Lin IF, Brown SL, Wright MR, Hammersmith AM. Antecedents of gray divorce: a life course perspective. J Gerontol B Psychol Sci Soc Sci. 2018;73(6):1022–1031. 10.1093/geronb/gbw164 PMC6093363 27986850

[38] Flood SM, Genadek KR, Moen P (2018). Does marital quality predict togetherness? Couples’ shared time and happiness during encore adulthood.

[39] Hazan C, Campa MI. Human bonding: the science of affectional ties: Guilford Press; 2013.

[40] Beraia I (2016). Evolution of Occurrence of Family and Marriage. J Adv Res Law Econ.

[41] Hill PB, Kopp J (2015). Editorial on the special issue "research on divorce: causes and consequences". Comp Popul Stud.

[42] Papp LM, Cummings EM, Goeke-Morey MC (2009). For richer, for poorer: money as a topic of marital conflict in the home. Fam Relat.

[43] Kanewischer EJ, Harris SM (2015). Deciding not to un–do the “I do:” therapy experiences of women who consider divorce but decide to remain married. J Marital Fam Ther.

[44] McNulty JK, Russell VM (2016). Forgive and forget, or forgive and regret? Whether forgiveness leads to less or more offending depends on offender agreeableness. Pers Soc Psychol B.

[45] Le B, Dove NL, Agnew CR, Korn MS, Mutso AA (2010). Predicting nonmarital romantic relationship dissolution: a meta-analytic synthesis. Pers Relat.

[46] Amato PR (2010). Research on divorce: continuing trends and new developments. J Marriage Fam.

[47] McNulty JK, O'Mara EA, Karney BR (2008). Benevolent cognitions as a strategy of relationship maintenance: "Don't sweat the small stuff".... But it is not all small stuff. J Pers Soc Psychol.

[48] Robles TF, Kiecolt-Glaser JK (2003). The physiology of marriage: pathways to health. Physiol Behav.

[49] Amato PR (2000). The consequences of divorce for adults and children. J Marriage Fam.

[50] Amato PR (2014). The consequences of divorce for adults and children: an update. Društvena istraživanja: časopis za opća društvena pitanja.

[51] Ernst E (2008). Chiropractic: a critical evaluation. J Pain Symptom Manag.

[52] Reggars JW (2011). Chiropractic at the crossroads or are we just going around in circles?. Chiropr Man Ther.

[53] Simpson JK (2012). The five eras of chiropractic & the future of chiropractic as seen through the eyes of a participant observer. Chiropractic Man Ther.

[54] Ernst E, Gilbey A (1312). Chiropractic claims in the English-speaking world. N Z Med J (Online).

[55] Young KJ (2017). Historical influence on the practice of chiropractic radiology: part I - a survey of diplomates of the American chiropractic College of Radiology. Chiropractic Man Ther.

